# Developing an Improved Ensemble Learning Approach for Predictive Maintenance in the Textile Manufacturing Process

**DOI:** 10.3390/s22239065

**Published:** 2022-11-22

**Authors:** Yu-Hsin Hung

**Affiliations:** Department of Industrial Engineering and Management, National Yunlin University of Science and Technology, Yunlin 64002, Taiwan; hungyh@yuntech.edu.tw

**Keywords:** predictive maintenance, data communication, ensemble learning

## Abstract

With the rapid development of digital transformation, paper forms are digitalized as electronic forms (e-Forms). Existing data can be applied in predictive maintenance (PdM) for the enabling of intelligentization and automation manufacturing. This study aims to enhance the utilization of collected e-Form data though machine learning approaches and cloud computing to predict and provide maintenance actions. The ensemble learning approach (ELA) requires less computation time and has a simple hardware requirement; it is suitable for processing e-form data with specific attributes. This study proposed an improved ELA to predict the defective class of product data from a manufacturing site’s work order form. This study proposed the resource dispatching approach to arrange data with the corresponding emailing resource for automatic notification. This study’s novelty is the integration of cloud computing and an improved ELA for PdM to assist the textile product manufacturing process. The data analytics results show that the improved ensemble learning algorithm has over 98% accuracy and precision for defective product prediction. The validation results of the dispatching approach show that data can be correctly transmitted in a timely manner to the corresponding resource, along with a notification being sent to users.

## 1. Introduction

The concept of Industry 4.0 involves digitalization, intelligentization, interconnectivity, and automation at the manufacturing site. Advances in digital transformation have reshaped the manufacturing industry into a highly cost-efficient business process. The common work order form includes the sequential number, product specification, operator ID, and quantity columns. The operators must fill the actual specifications of the finished product into the work order paper form while completing the product in the conventional production line. The human-written content or the loss of documents may lead to incorrect information and missing information, respectively. However, the paper form also increases the difficulty of data sharing. The inspector usually views the real textile product without any assistance data, because the inspector rarely refers to the content of the work order paper form. IoT-related applications are widely applied at manufacturing sites. Such types of applications include internet-connected devices and software applications that aim to wire the smart objects within cyber physical systems (CPS) for industrial applications. In recent years, paper forms have started to be transformed into electronic forms (e-Forms) through IoT-related applications. This proposed use case utilizes the computerized sewing machine, and the specifications of the finished product are automatically recorded in the work order e-Form. During the manufacturing process, industrial data can be obtained within the CPS environment at the manufacturing site. The collected data can create more value in the manufacturing process by learning from the data, exploring the valuable information, and sharing it with others. Predictive maintenance (PdM) is a technique used to predict anomalies in the work process and potential equipment failure; thus, the anomalies can be addressed before failure occurs. The core principle of Industry 4.0 is value creation; the usage of the industrial data in an economical and efficient way to add value to the manufacturing site. The manufacturing data can be applied in data analytics to obtain useful information, and human operation can be assisted by the analytics results in the specific manufacturing process. For instance, although the use case for the new screw machine was to record the work order e-Form, the data were rarely extensive. The inspectors still view the textile product according to their experience without referring to the data recorded by the machine. Because the production and inspection workflows are separate, it is difficult for them to benefit from data sharing. To deal with the data utilization challenges in the textile product manufacturing process, the key objectives are presented in two aspects: data analytics and system implementation. Data analytics is used to achieve the goal of intelligentization. This study applied ensemble learning approaches (hereinafter ELAs) to forecast unqualified product. The author proposed a hybrid approach consisting of a bagging and boosting ensemble method to improve computation performance. The input dataset is from an e-Form (work order form) that included six attributes concerned with product size and employee ID. The input data were preprocessed using data balance and feature selection methods. The preprocessed data were imported into the ELA model to predict defective label data. The analyzed outcome is a binary classification of product data (defective or non-defective label). System implementation used cloud computing technologies to achieve the goal of automation. Cloud service providers supply a free third-party resource with limited service. Such providers (e.g., Google, Sendgrid) can send a limited quantity of emails per day for registered users with different account levels. If the quantity of emails exceeds the daily constraints, users may lose their email notifications. Consequently, using a single resource may run the risk of interrupting email notifications if usage exceeds the allotted usage amount; thus, multi-resource maintenance was used to arrange and backup resources to maintain uninterrupted notifications. The author proposed a data dispatching approach that integrates manufacturing data and the third-party emailing application. The data and analytics can be automatically communicated from machine to human in a timely manner with the correct content and the correct emailing resource. The aims of this study are as follows:

- To intelligenize the quality management by forecasting the defective class of product using ELAs.
- To enable the automatic notification by developing the proposed resource dispatching approach in data communication.
- To enhance the efficiency of PdM by integrating the above mentioned data analytics and systems in the textile product manufacturing process.

The remainder of this study is organized as follows: Section 1 introduces the research background, the current issues, and the research objects. Section 2 reviews the PdM-related literature and integrates the related technologies. Section 3 describes the procedure of data analytics using the proposed approach and the system design of data communication. Section 4 describes the experiment procedure, the used dataset, and evaluation criteria. Section 5 presents the results of our analyses. Finally, Section 6 concludes this study, describing the study’s significance and limitations and providing suggestions for future work.

## 2. Related Work

This section presents a survey of related studies involving PdM in the manufacturing industry. Initially, this study relied on the Publish or Perish system to gather all relevant studies. The time trend and disciplinary distribution of PdM-related topics were analyzed, which retrieved 126 relevant studies from 2010 to 2022/8. The total number of citations of the relevant studies is 1450 over the twelve-year span. The average number of citations per year is 120.83, and the average number of citations per paper is 11.51. Next, this study manually sorts the studies according to their average number of citations per year and total number of citations and surveys in the relevant papers. “Cyber physical system”, “Internet of things”, “data-driven”, “data analytics”, and “cloud computing” are high-frequency keywords for PdM-related topics in the manufacturing industry. The current approach for PdM in manufacturing are classified into sensors, network, mobile integration, cloud systems, mathematics, data mining, statistics, and machine learning.

Value-addition and co-creation are trends in the current development of PdM, and quantitative and qualitative methods have been applied to PdM. Some researchers indicated the challenges encountered when building a data value chain for the PdM of a grinding machine in 5G-enabled manufacturing [1]. Simulation models and a digital twin concept were used to predict the remaining useful lifetime (RUL) of machinery equipment [2]. Strategy management was applied to PdM of product quality to reduce manufacturing costs [3]. To prioritize PdM component-sets, Han et al. proposed a mission reliability-oriented RUL prediction approach and average maintenance cost calculation [4]. Using a CPS and big data analytics, He et al. proposed a cost-oriented dynamic predictive maintenance strategy [5]. Gutschi et al. used data mining and random forest (RF) methods to forecast the probability of machine breakdown in specified future time intervals [6]. The improved Monte Carlo approach has been applied to identify the health factors of equipment in semiconductor manufacturing [7]. Annamalai et al. estimated the faults that could occur in machines and determined the timing in which a critical situation could occur [8]. Real-time data and predictive analytics algorithms for PdM decision-making were proposed in [9]. The system development and machine learning were applied in motor PdM, through the mobile agent and a signal processing algorithm for remotely monitoring and controlling [10]. Big data storage, cluster setting, and principal component analysis (PCA) were applied to develop the system for fault prediction and pre-diagnosis [11]. The related researchers overviewed the machine learning approaches applied in PdM [12]; the survey results indicated that support vector machine (SVM), RF, and artificial neural network (ANN) were the most used in PdM [12]. Supervised learning approached such as SVM and logistic regression (LR) rely on data output from the previous experience, thus the computation performance can be optimized by using experience. An ANN and a SVM were employed to predict tram-track gage deviation [13]. SVM creates a hyperplane and uses the kernel trick to find the best classification. LR uses different decision boundaries with different weights to find the optimal solution. SVM has a faster computation time than LR. However, SVM can not easily explain the classification in terms of probability. Meanwhile, SVM, RF, and gradient boosted machine (GBM) were used in in machine prognostics and diagnostics [14], and K-means was used to maintain a selective laser melting machine [15]. Moreover, PCA and K-means were applied in the maintenance of industrial machines [16]. Gohel et al. utilized SVM and logistic regression to pre-diagnose and monitor power consumption in the nuclear infrastructure [17].

ELAs are the predominant supervisory learning approach and use multiple learning algorithms to obtain superior predictive performance. The results of associated research have demonstrated that ELAs typically outperform any constituent learning algorithm alone [12]. While processing a dataset that consists of nonlinear and linear data, ELAs can assemble different models to handle such data. ELAs have a sophisticated computational approach in comparison to individual models and can reduce variance to solve data over-fitting and under-fitting. GBM, RF, XGBoost, and light gradient boosted machine (LightGBM) are the approaches used to assemble the tree model, offering superior classification performance in labeled data analytics. XGBoost grows the trees with the depth-wise method, LightGBM grows trees with the leaf-wise approach, and RF grows the tree with the random approach. The main disadvantage of RF is the higher complexity compared with the LightGBM and XGBoost. LightGBM and XGBoost will most likely win in terms of performance and speed compared with RF. Properly tuned LightGBM has better classification performance than RF. LightGBM is based on the histogram of the distribution. LightGBM requires lesser computation time and lesser memory than RF, XGBoost, and decision jungle. Taking PdM equipment as an example, GBM, RF, XGBoost, and neural network approaches were used to forecast the RUL of woodworking machines [18]. ELAs have also been used for assessing production line maintenance [19]. The supervisory learning method has been widely applied in industrial applications of PdM in the manufacturing industry, with a good predictive performance in manufacturing data analytics. Supervised machine learning has excellent performance in predicting numerical data with given labels. Multiple forms of manufacturing data (e.g., equipment configuration data, e-Form data) being assigned specific labels and data attributes obviously leverages data output from previous experience, and such data are suitable for supervisory learning. Deep-learning approaches can include supervised, unsupervised, and semi-supervised learning which have started to be applied in PdM in recent years. Deep-learning is appropriate for analyzing large datasets, complex data attributes, and various data forms, such as images and signals. In comparison to deep-learning approaches, ELAs require less computation time and lower computing environment specifications in labeled data analytics.

To summarize, this section provides an overview of the PdM used in the manufacturing industry. PdM for industry 4.0 is an advanced concept for predicting potential abnormal events by analyzing data from various sources, such as the sensor, the machine, the system, and the e-Forms, to identify patterns and predict issues before failure happens. Due to the high costs of maintaining today’s sophisticated and complex equipment, it becomes necessary to improve the efficiency of modern maintenance management; thus, some studies aim to maintain the machine [8,15,16,20,21]. Some researchers have proposed a mathematical model and data mining model to support a learner model for accessing and processing data [2,3,6,7,10,13,14,15,19]; thus, in a different field, such as the edge-cloud environment, the maintenance strategy can be predicted. ELAs are the integration module that aggregates the multiple model for the enhancement of predictive performance. This study also investigates an ensemble learning-based analytics method to achieve the goal of PdM. PdM research has started to attract much attention from researchers. Li et al. integrated the member algorithms to predict the RUL of aircraft engines and aircraft bearings, and the results showed that the ensemble learning prognostic was less able to predict error [22]. Wu et al. proposed the RF-related algorithms that aggregate the multiple decision trees to forecast the tool wear in the dry milling process [23]. Some researchers used an RF-related algorithm to forecast software defects [24]. The boosted decision tree is used to predict pipe failures [20]. Shehadeh et al. investigated a modified decision tree, LightGBM, and extreme gradient boosting (XGBoost) regressions to predict the residual value of construction equipment [21]. The results demonstrate that the ensemble learning solution offers a convenient tool for users to analyze the manufacturing data and make a maintenance strategy. The manufacturing data this study used is from e-Form, thus, the data has defined attributes. Additionally, the defective product detection quite heavily relied on previous experience or observation. Therefore, this study used the ELA to predict the defective class of product. This study also developed a cloud application for the further use of data. 

## 3. Methodology

**Figure 1:**
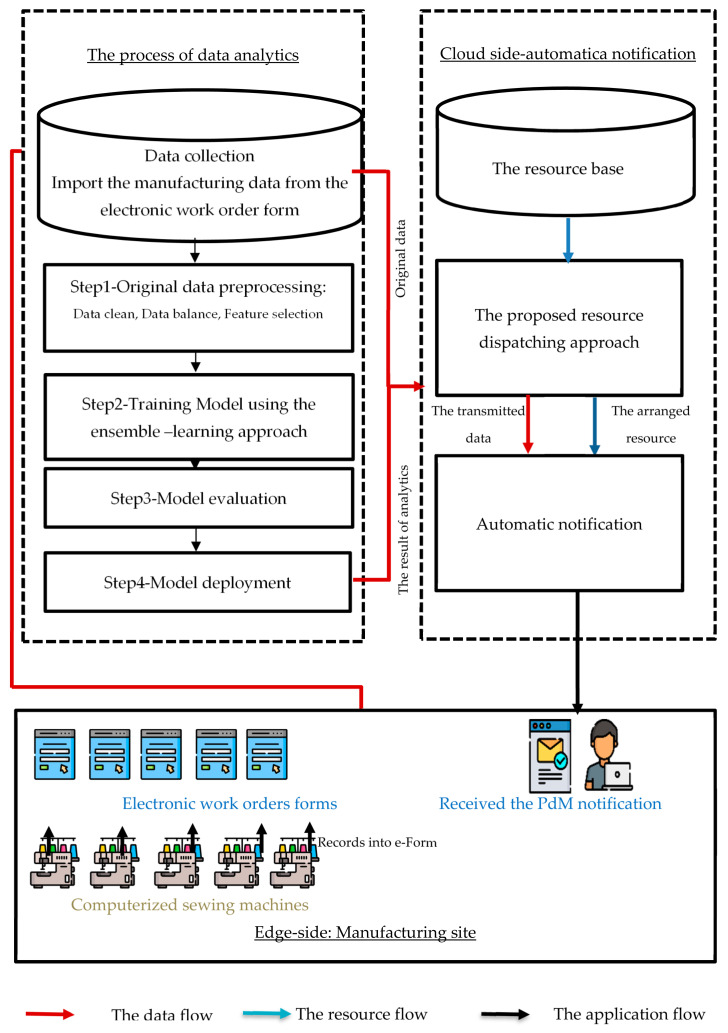
The flowchart of the data analytics in this study.

This study proposed an overall workflow based on data from computerized sewing machines at the manufacturing site, edge data transmitted to the cloud for data analytics, and automatic notification of PdM implementation (Figure 1). Previous studies have shown that statistical methods, mathematics, and data mining machine learning have been used to address the PdM issue. In some cases, machine learning approaches are extremely accurate. The ELA was used in this study for PdM in the textile manufacturing process. The input was the data collected from the e-Form, and the predictive target quality of textile products is defective or non-defective. In this study, the manufacturing data were processed according to the standard data workflow process. The “knowledge discovery-in database” involves data collection and preprocessing, modeling, model evaluation, and deployment (Figure 1). Therefore, it can provide better insights into processes while also allowing for the exploration of the value of the imported data. This study makes the most of the original data, and the analytics results are transmitted to the cloud via automatic notification. Prior to the failure event, the user can obtain the notification and perform early maintenance execution. This study proposed a resource dispatching approach; the transmitted data was applied in the arranged third-party resource to send the notification for a user.

### 3.1. Data Preprocessing

The original dataset needs to be preprocessed, such as missing a value handle. The data from the manufacturing case and the blister packing machine case need to be processed. Data imbalance means that the sample size of data with one class outnumbers the others by a large proportion. Most of the real datasets in the manufacturing field have the problem of imbalanced proportion. Both of the cases need to be preprocessed for data balance. The synthetic minority oversampling technique (SMOTE) was used in this study to balance the proportion of data from each class. SMOTE is an oversampling method proposed by Chawla et al. that is based on the k-nearest neighbors in the solution space, as determined by Euclidean Distance between data points [25]. The SMOTE method’s procedure:

■ Step 1: Explore the minority class input data point.
■ Step 2: Find the k-nearest neighbors of explored input data point.
■ Step 3: Select one of these neighbors’ point, and placing a new point on the path connecting the point under consideration and its chosen neighbor.
■ Step 4: Repeat Steps 1 and 2 until the terminal condition (data had been balanced).

After balancing the data proportion, the features were selected from the preprocessed data though the data balance method and mutual information (MI) estimation. The MI is a score that measures variable mutual dependencies and is useful in feature selection because it maximizes MI and joint distribution in datasets with many dimensions (See Equation (1)).

(1) $$\mathrm{MI}(X;Y)=\underset{y\in Y}{\sum }\underset{x\in X}{\sum }prob\left(x,y\right)\log(\frac{prob\left(x,y\right)}{prob_{1}\left(x\right)prob_{2}\left(y\right)})$$

where MI(*X*;*Y*) represents the MI between the variable *X* and variable *Y*. *Prob*(*x*) and *prob*(*y*) refer to the probability distribution of *x* and the probability distribution of *y* separately. The *prob*(*x*,*y*) represents the joint probability distribution of *X* and *Y*.

### 3.2. Preliminaries Ensemble Learning Model

This study used the emerging ELA to predict whether a product is defective or non-defective. Ensemble learning is a concept of collaborative learning using more than one single model, and single models can be assembled into training models with different types of ensemble methods. Ensemble methods are classified into two types, “boosting” and “bagging”. Breiman proposed the “bagging” concept [26]. Bagging is a method in which a model is trained many times using different subsets from the training data [27]. The final output prediction is then averaged across the predictions of the sub-models [26]. The concept of bagging is to decrease the variance of the classification errors to reduce the accuracy of the prediction model. Using the “bagging” method, single decision trees can be aggregated to form an RF and decision jungle. The boosting method was developed by [28]. In comparison to the “bagging” approach, the “boosting” method is an iterative approach in which the first model is trained on the entire training dataset and adjusts the weight of an observation based on the previous result. The previous result can be reinforcement trained by applying a higher weight to the stronger observations [27,28]. Boosting was applied in LightGBM for enhancing the prediction performance via the iterative modification. The RF, decision jungle, and LightGBM are the preliminary models this study used in the data analytics model. This study proposed the reinforcement training mechanism to improve LightGBM.

#### 3.2.1. Random Forest

RF is proposed by Ho [29], and Breiman extensively developed the RF approach [30]. RF is a popular and useful approach, which is widely applied in different fields. RF ensembles are made up of multiple decision trees. Each individual decision tree in the RF has a class prediction, and the prediction result with the highest frequency in the overall model becomes the final model’s prediction result. The classification performance of each individual decision tree is important for the overall RF model performance. Adequate evaluation criteria for the decision tree model are essential for an RF model. Gini impurity means the classification performance of decision tree splitting. Equation (1) is the formula of the Gini impurity used to estimate the probability of a selected feature would be incorrectly classified when selected randomly. The RF uses Gini impurity to determine the tree splitting [31].

(2) $$\mathrm{Gini}=\overset{L}{\underset{i=1}{\sum }}Prob\left(i\right)\times \left(1-Prob\left(i\right)\right)$$

where, L represents the total number of labels in the subset of data, and i is a selected label out of L. *p*(*i*) represents the probability of selecting label *i*.

#### 3.2.2. Decision Jungle

The decision jungles proposed by Breima are rooted directed acyclic graphs (DAG) [32]. The main difference between a conventional decision tree and a decision jungle is the quantity of training paths from the root to the leaves. A decision jungle can have multiple paths from the root to each leaf, whereas only one path is allowed in the decision tree. A decision jungle optimizes the tree build; the tree generation, such as node merging and splitting, is based on the minimization of the same objective function. In the training procedure, the path of the DAG was built independently, and one level of the DAG can be created at one time. Equation (2) is the objective function [32]. The objective function minimizes the child node i objective $\left\{r_{i}\right\},\left\{l_{i}\right\}$ and split objective $\left\{\alpha_{i}\right\}$. The objective function is associated with the current level of the DAG is a function of {S_j_}_j∈N_. Where *Entropy* (S) is the Shannon entropy of the class labels in the training instances. The formula of S shows the set of instances from node i that travel through its left branches (L) and right branches (R).

(3) $$\underset{\left\{\alpha_{i}\right\},\left\{r_{i}\right\},\left\{l_{i}\right\}}{\min}E(\left\{\alpha_{i}\right\},\left\{r_{i}\right\},\left\{l_{i}\right\})=\underset{\left\{\alpha_{i}\right\},\left\{r_{i}\right\},\left\{l_{i}\right\}}{\min}\underset{j\in N_{\mathrm{child}}}{\sum }\left|S_{j}\right|{\mathrm{Entropy}(S}_{j})\;\;s.t.\;S_{j}\left(\left\{\alpha_{i}\right\},\left\{r_{i}\right\},\left\{l_{i}\right\}\right)=\left[\underset{i\in N_{parent\;s.t.l_{i}=j}}{\cup }S_{i}^{L}\left(\alpha_{i}\right)\right]\cup^{\;}\left[\underset{i\in N_{parent\;s.t.r_{i}=j}}{\cup }S_{i}^{R}\left(\alpha_{i}\right)\right]$$

where, $N_{\mathrm{parent}}$ and $N_{\mathrm{child}}$ represent the set of parent nodes and a set of child nodes, separately. $\left\{\alpha_{i}\right\}$ represents the parameters of the split feature function for the parent node $i\in N_{\mathrm{parent}}$. Assumed that the child node $j\in N_{\mathrm{child}}$. Si is the set of labeled training instances that reach node i. Let $l_{i}\in N_{\mathrm{child}}$ is the current assignment of the left edge from the parent node $i\in N_{\mathrm{parent}}$ to a child node, and similarly for the right edge $r_{i}\in N_{\mathrm{child}}$ [32].

#### 3.2.3. eXtreme Gradient Boosting (XGBoost)

**Figure 2:**
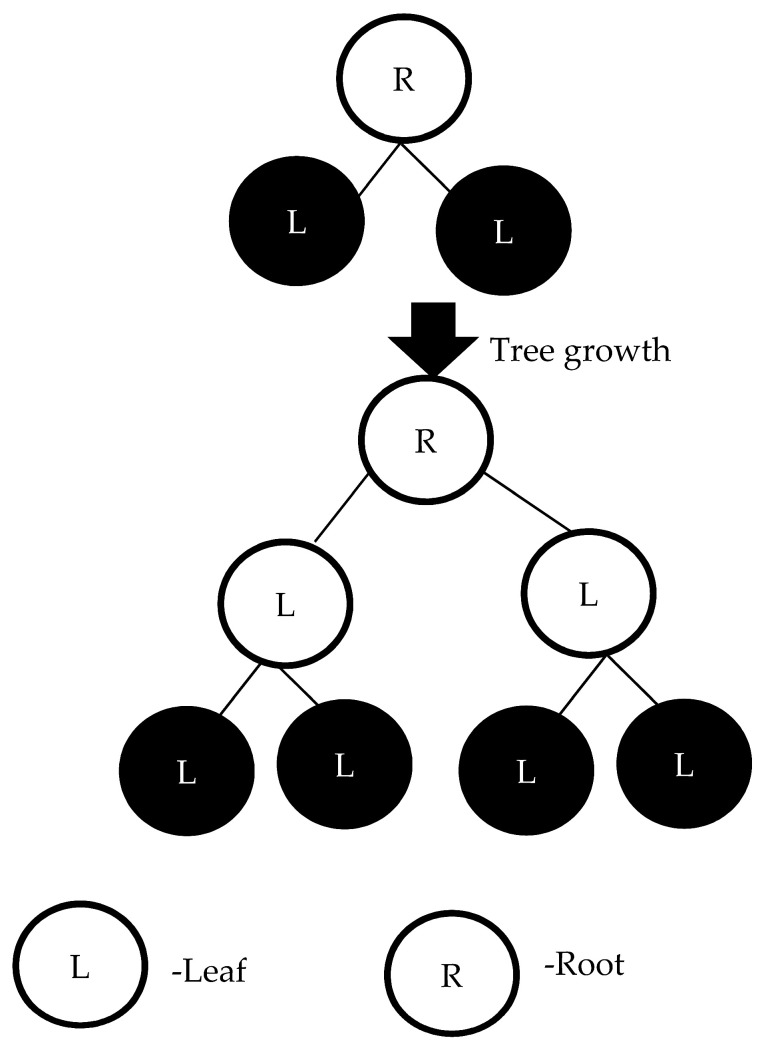
The tree grown level-wise.

XGBoost is based on a gradient boosting framework [33] and improves upon the speed and computational performance of original gradient boosting models. The XGBoost model sequentially assembles multiple decision trees according to weight. Such weight estimation is critical to the assembly of XGBoost. Model training is performed sequentially, adjusting the weight of variables according the incorrect prediction and forwarding the variables to the next decision tree. After this sequential training, the ensemble model becomes a strong model. XGBoost constructs trees using a level-wise method, meaning that a tree is grown level by level (see Figure 2).

#### 3.2.4. LightGBM

**Figure 3:**
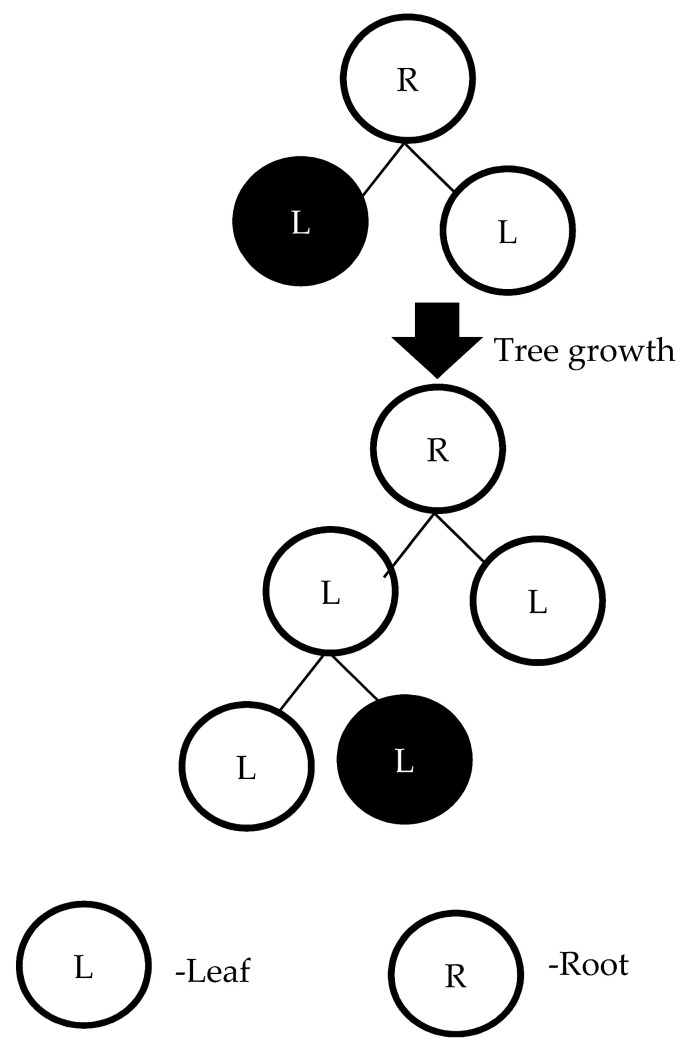
The tree grown leaf-wise.

Boosting was applied in the ensemble method to improve accuracy with a small risk of reduced coverage. The gradient boosting decision tree approach is used to combine many individual decision trees and adjust the errors of the previous tree to form a strong model. All the trees are sequentially connected together. Each individual decision tree tries to minimize the error of the previous tree in the ensemble model. Due to this sequential connection, the gradient boosting decision tree has high accuracy. The outcomes of each tree were integrated to evaluate and find the optimal prediction result. Ke et al. proposed LightGBM. LightGBM is an ensemble of gradient boosting decision trees [34]. The LightGBM is used to maintain the high accuracy of the model while decreasing the memory usage of the computing environment. Figure 3 depicts how LightGBM grows trees using the leaf-wise method, which selects the leaf with the greatest decrease of loss based on the histogram, rather than checking all previous leaves for each new leaf [35].

#### 3.2.5. Proposed Method: Reinforcement Training for LightGBM

**Figure 4:**
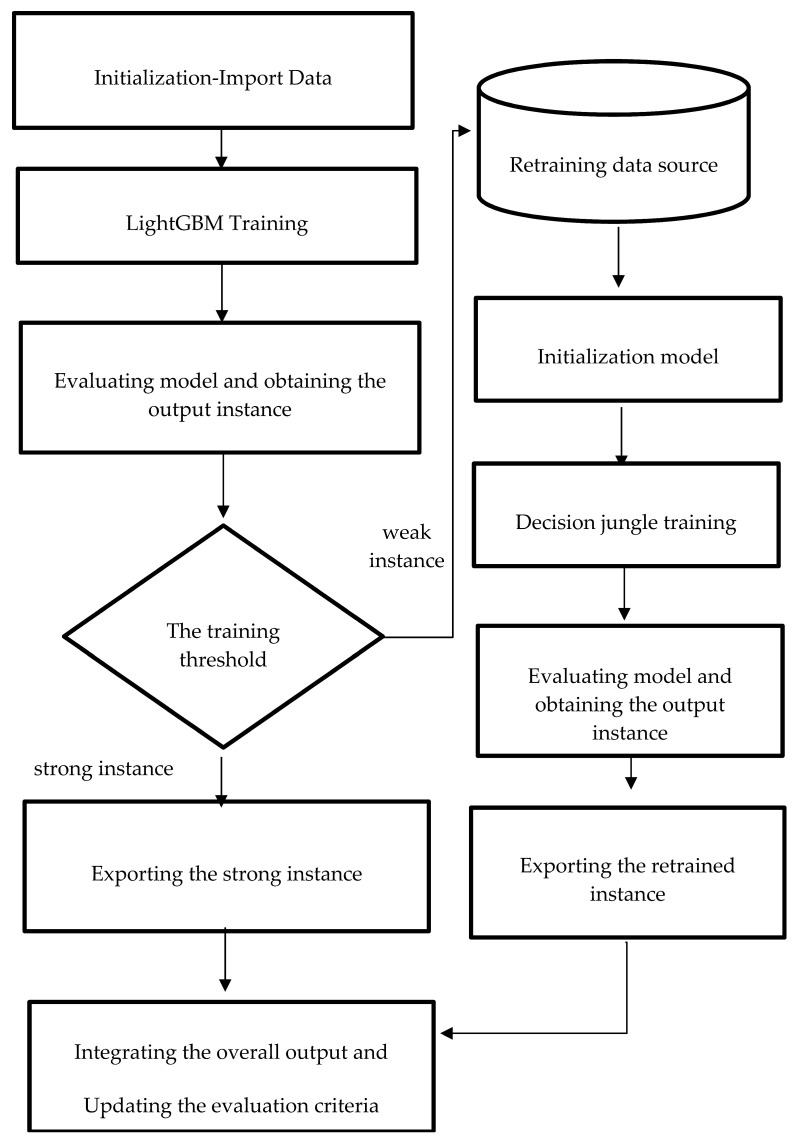
Procedure of the proposed method.

Boosting is an iterative approach in which the latter tree corrects for the errors of the previous tree. The outcomes of each tree were integrated to evaluate and find the optimal prediction result. This study improves the model’s efficiency using a reinforcement training mechanism, defined as a weaker observation data source that should be retrained with the bagging method. The bagging approach attempts to address the over-fitting complex issue by training many “strong” learners in parallel. A strong learner is an unconstrained model, and the reinforcement training mechanism improves accuracy by expanding the rebuild solution space for weaker observations to explore. This study used a decision jungle to retrain the weaker observation data source from the trained boosted decision tree model. Figure 4 shows a flowchart of the proposed method. The processed data were imported into the LightGBM model in the initial stage. Then, the trained model was evaluated to determine the weaker instance (meaning the instance with lower accuracy) and the stronger instance (meaning the instance with higher accuracy). The weaker instance is imported to the retraining data source, which is fed forward to the decision jungle for reinforcement training. If the retained result is better or similar to the initial model, then the retrain model integrates with the initial model. The result of the strong instance of the initial model and the retrain result are ensembled as the overall output. Therefore, the proposed method was applied to data analytics for the PdM problem: the textile use case. This study compared the predictive performance of the new approach, single- and ensemble-based modules, using the conventional decision tree algorithm, RF, decision jungle, and LightGBM.

### 3.3. Data Communication Using the Proposed Resource Dispatching Approach

**Figure 5:**
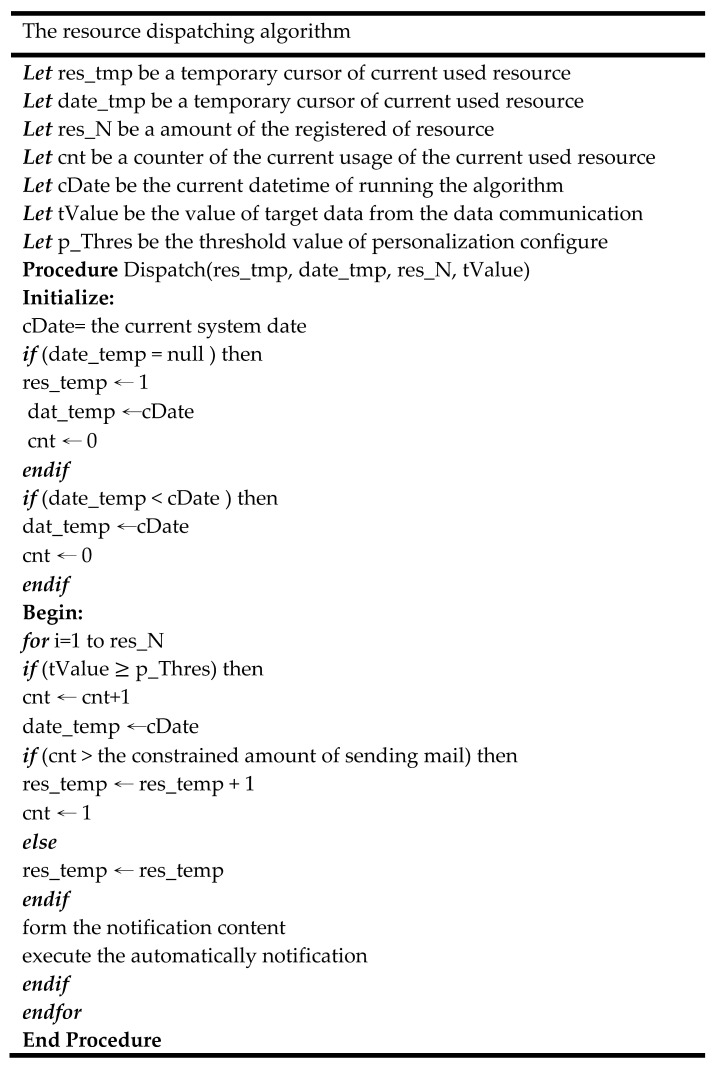
Procedure of industrial data analytics from import to deployment.

The further use of data is important for data-driven digital transformation in the manufacturing site. With the rapid development of IoT, IoT-related applications have become increasingly important. Various types of third-party resources have been widely applied in different value co-creation applications. A user can access third-party resources with limited service quotas after that user registers on the official platform. Taking emailing services as an example, the common registered user has a limited amount of free email send services per day. The notification may not be successfully sent to the user when the number of sent emails has exceeded the constrained amount of resources. To improve the efficiency of using the resource, this study proposed the resource dispatching approach and used the proposed algorithm to arrange the resource in the resource database. Figure 5 is the pseudo-code of the proposed approach. This study uses the temporary cursor and counters to implement the algorithm. In the pre-execution stage, this study pre-builds the resource database and the properties which recorded the information of the registered information, the resource usage, the updated date of resource dispatching, and the personalized notification threshold. The parameters included the amount of the registered resource from the resource base; the above parameters were imported into the algorithms. In the start stage, the algorithm provided the start for the first use (there is no existing date temporary cursor) and the date time start for the resource dispatching cross the next day. For the first use, the parameter is set as the initial value. According to the guidelines from the third-party service provider, the constrained amount of sending mail was recovered as zero on the next used day. For the date start, the counter also needs to be set as zero. After parameter start, then start to enable the algorithm main procedure. The first step is updating the time cursor as the current system date time. The second step is to estimate that the target value is achieved by the notification threshold. The function of automatically notification enabled when the data value exceeded the threshold value. The third step determines whether the current resource arranges to the next resource. The resource can still be used if the usage is on the range of the constraint amount of emailing usage. The resource was arranged to the next resource during the current usage of the currently used resource is exceeded the constraint amount of emailing usage. Finally, if the notification condition existed, and the using resource had been arranged, the arranged resource was applied in the notification by sending the email service with the customized content.

## 4. Experiment

**Figure 6:**
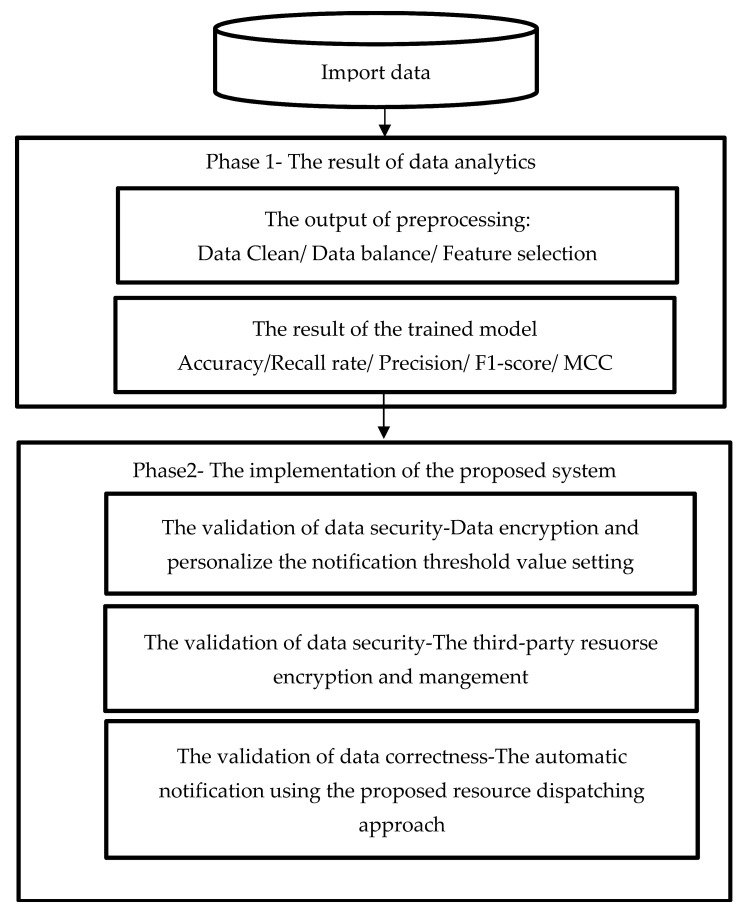
The flowchart of the experiment.

In this study, the e-Forms concerned with the work order form in the real textile production site was used as a use case. The use case in this study aimed to forecast the current quality of the finished product (e.g., defective or normal) in the textile manufacturing process. This case, which used the computerize sewing machine to record the specification of the finished product, operator into a work order form. The content of the work order form was saved in the database. Figure 6 shows the flow of the experiment. The experiment consisted of two parts: data analytics and the implementation of the system design. On the viewpoint of data analytics, the dataset initially need be preprocessed. Next, the preprocessed data are imported into the proposed improved ensemble learning based model. On the viewpoint of system implementation, the data can be integrated into the cloud system for further use, such as early notification using the proposed dispatching approach.

### 4.1. Dataset and Computation Enviornment Description

The listed sources were used for data analysis in order to address the problem of early failure diagnosis in the textile manufacturing process. The experiment data this study used is the quantified data. It needs to be collaboratively used with the central processing unit (CPU); specifically, random access memory and the hard disk were used to support the data analytics. Table 1 demonstrates the specification of the experiment environment. The experiment was run in an environment that consisted of the processing, storage, and graphic process unit. In this study, the data are collected from the digital work order form. The data comprised 500 instances with six production attributes (Table 2).

### 4.2. Evaluation Criteria

**Figure 7:**
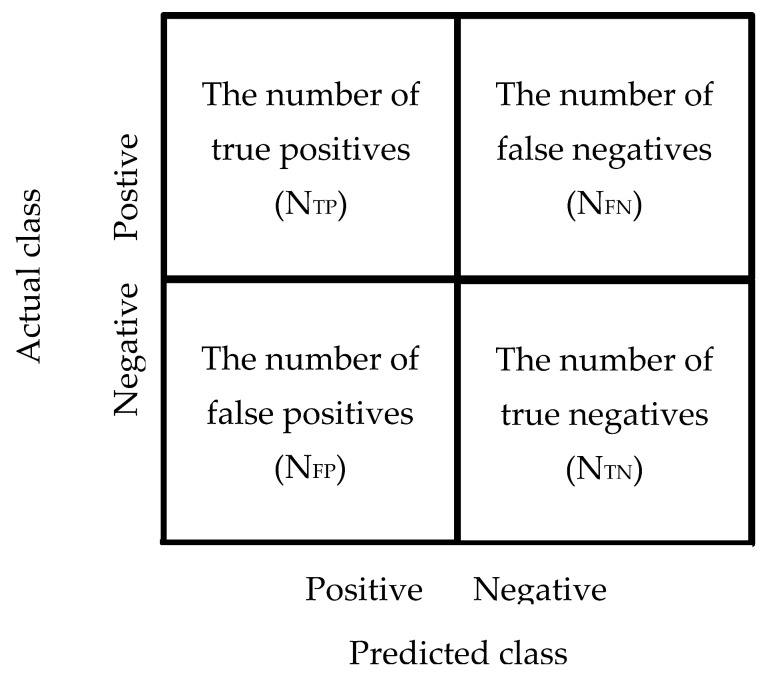
The confused matrix and the element classification.

The evaluation item based on the two-dimension confused matrix, prediction and actual results, and matrix can be classified into four elements (Figure 7): false negatives (FN), true negatives (TN), false positives (FP), and true positives (TP). The element of the confused matrix is used to estimate accuracy, precision, recall rate, F1-Score, and Matthews correlation coefficient (MCC) for evaluating prediction performance. The evaluation criteria are described below.

#### 4.2.1. Accuracy

The accuracy rate refers to the proportion of predictions that are correct. The accuracy rate is estimated by the aggregation of correct assessments divided by the sum of all assessments. The accuracy rate is the degree of closeness to the correct value, which can be regarded as the degree of veracity. The accuracy formula parameters include the number of false negatives (*N_FN_*), the number of true negatives (*N_TN_*), the number of false positives (*N_FP_*), and the number of true positives (*N_TP_*).

(4) $$\mathrm{Accuracy}=\frac{N_{\mathrm{TP}}{\;+\;N}_{\mathrm{TN}}}{N_{\mathrm{TP}}{\;+\;N}_{\mathrm{TN}}\;+\;N_{\mathrm{FP}}{\;+\;N}_{\mathrm{FN}}}$$

#### 4.2.2. Precision Rate (Positive Predictive Value, PPV)

Accuracy and precision are the two main criteria for model evaluation. The precision rate means the proportion of accurate predictions for the positive assessment. The precision rate value is estimated as the proportion of actual positives divided by the sum of all positive assessments. The precision rate can be regarded as the degree of reproducibility. The formulas of precision are expressed as:

(5) $$\mathrm{Precision}\;\mathrm{rate}=\frac{N_{\mathrm{TP}}}{N_{\mathrm{TP}}{\;+\;N}_{\mathrm{FP}}}$$

#### 4.2.3. Recall Rate (True Positive Rate, TPP)

The recall rate is a statistical measure of the classification function’s performance. The recall rate value is estimated by the proportion of actual positives identified correctly that should have been predicted as positive.

(6) $$\mathrm{Recall}\;\mathrm{rate}=\frac{N_{\mathrm{TP}}}{N_{\mathrm{TP}}\;+\;N_{\mathrm{FN}}}$$

#### 4.2.4. F1-Score

The F-score estimation is the combination of precision rate and recall rates in the model. The F1-score refers to the harmonic mean of the model’s precision rate and recall rate.

(7) $$\mathrm{Recall}\;\mathrm{rate}=\frac{2\times \left(Precision\;rate\times Recall\;rate\right)}{Precision\;rate+Recall\;rate}$$

#### 4.2.5. Matthews Correlation Coefficient

MCC is the evaluation criterion concerned with the classification performance of a confused matrix. MCC uses actual and predicted values to estimate the Pearson product moment correlation coefficient. The formulas of the precision rate are expressed as Equation (8). The range of the MCC value is from −1 to 1. The value of −1 presents the great disagreement between actual and predicted values, and 1 denotes the great agreement between actual and predicted values.

(8) $$\mathrm{MCC}=\frac{N_{\mathrm{TP}}{\;\times \;N}_{\mathrm{TN}}{\;-N}_{\mathrm{FP}}{\;\times \;N}_{\mathrm{FN}}}{\sqrt{\left(N_{\mathrm{TP}}{\;+\;N}_{\mathrm{FP}}\right)\left(N_{\mathrm{TP}}{\;+\;N}_{\mathrm{FN}}\right)\left(N_{\mathrm{TN}}{\;+\;N}_{\mathrm{FP}}\right)\left(N_{\mathrm{TN}}{\;+\;N}_{\mathrm{FN}}\right)}}$$

## 5. Results

This study describes the results from the viewpoint of data analytics and the viewpoint of the system implementation. This study demonstrated the predictive results of the proposed approach and ELAs for the use case of data analytics. The effect of ensemble models was also considered, and this study investigates the relevance between the ensemble models and the single model in the classification performance. This study demonstrated how the data can communicate using the proposed resource dispatching approaches and correctly use the corresponding resource based on the constraints of the third-party resource on the system implementation.

### 5.1. The Aspect of Data Analytics-the Result of the ELAs in PdM

**Figure 8:**
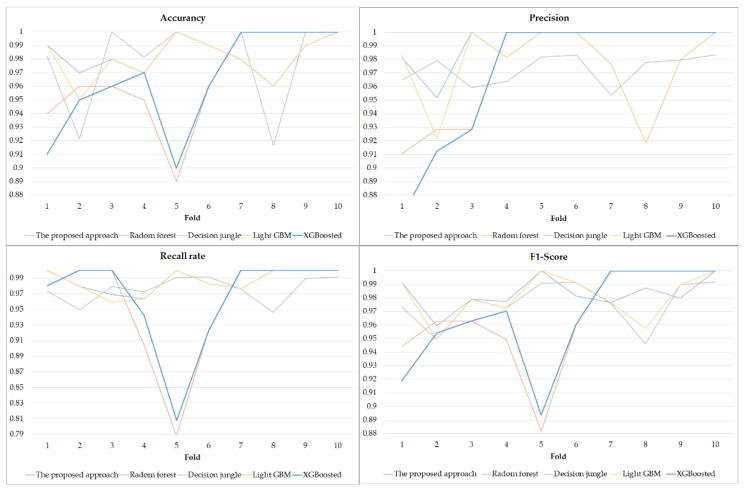
The results of 10-fold validation.

In the beginning, the original data were preprocessed using data cleaning to remove an unnecessary column. Then, the SMOTE algorithm was used to generate the new data according to the original data for data balance processing. After the data balance processing, the quantity of data with the no-defective label was enlarged from 460 to 480, and the quantity of data with the defective label was enlarged from 40 to 520. The amount of new data was 1000, and forty-eight percent of the new data had a non-defective label, and fifty-two percent of the new data had a defective label. This study used the preprocessed data to predict the defective product in the use case. In this study, K-fold cross-validation is used to evaluate the trained model to avoid the possible bias from data split. Cross-validation is a statistical approach that is widely applied to estimate the performance of machine learning models. K-fold cross-validation is a common method for comparing and selecting a model for a given predictive model by resampling the dataset recursively. The concept of K-fold cross-validation is to recursively split the original set into several subgroups, and to compute an average value over the partitions. This study used 10-fold cross validation. 80% of the dataset is used as a training dataset, 10% of the dataset is used as a validation dataset, and 10% of the dataset is used as a training dataset. The variance and standard deviation result of evaluation criteria over repeated runs is presented in Table 3. Figure 8 shows the result of each fold. Due to pseudorandom mechanisms that existed in the machine learning approach, the above results assess the variability of the machine learning approaches within 10-fold cross validation. The overall results presented low variability, which means that the ELAs could better predict the class of the product based on sample data. Table 4 showed the mean of the accuracy, precision, recall rate, F1-score, and MCC of the use of ensemble learning models within 10-fold cross validation. The results using the proposed model showed that the accuracy and the precision rate were 0.983 and 0.979, respectively. The decision jungle result showed that the accuracy and precision rate were 0.980 and 0.980, respectively. The RF had an accuracy of 0.966 and a precision rate of 0.977. The LightGBM had an accuracy of 0.981 and a precision rate of 0.976. The results showed that using LightGBM and decision jungle had similar predictive outcomes (Table 3). The ELAs applied in the experiment were based on a similar training framework. All of the ELAs used have adequate classification performance (accuracy, F1-Score, and MCC) and outstanding discrimination of the data with a defective label (precision and recall rate). The RF and XGBoost approach has more computation time than other machine learning approaches, the computation time using the proposed approach is in between the other approaches. In summary, the result obtained using the proposed method was compared with that of the LightGBM and decision jungle. Furthermore, the obtained results indicate that the ELA achieves greater than 98% accuracy in the use case.

### 5.2. Aspects of System Implementation—Notification for PdM

**Figure 9:**
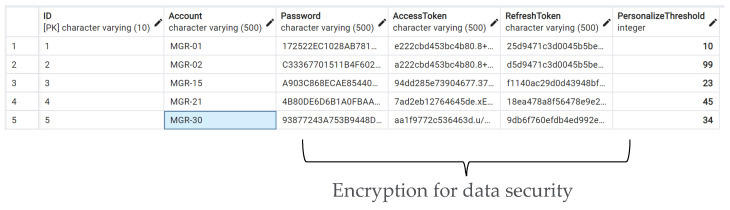
The results of the personalized notification setting.

The trained model was deployed as a RESTful application programming interface (API), which could be used in edge-based or cloud-based analytics for PdM. The data analytics process is fully automated after the client sends the analytics requirement to the server via the API. The submission is detected by the server-side process and automatically imported to the trained model according to the API message. The server-side response to the analytics results are sent to the client side, which sends an API request for automatic notification. The data communication involved with the original data, or the analyzed data in this study to meet the different requirements notification. The notification can be normalized and generalized; this study provides the personalize threshold value for notification and the default value for general use. Figure 9 demonstrates the encoded personal identification and the corresponding notification threshold value. This study encrypted the API user password using the MD5 algorithm. For the maintenance of API security, this study provided the encrypted API token for the user to access the services after the user’s login details passed validation. When the user’s access token has expired past the authorized period, this study provided a refresh token for the user to obtain a new access token instead of logging in again.

**Figure 10:**
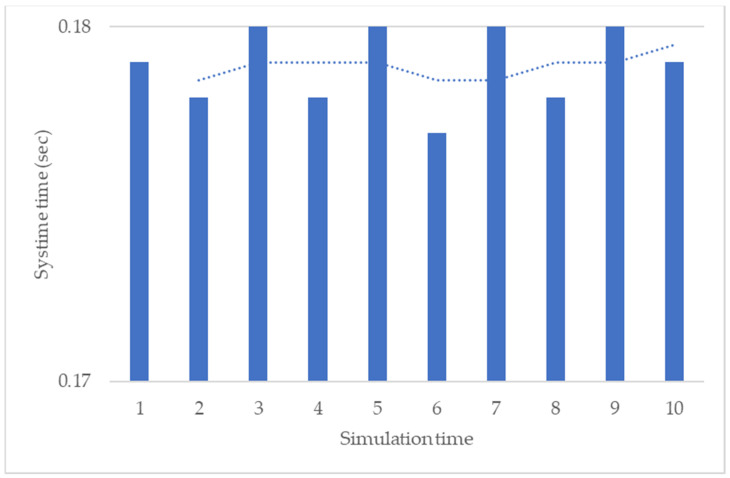
The results of the resource pool.

**Figure 11:**
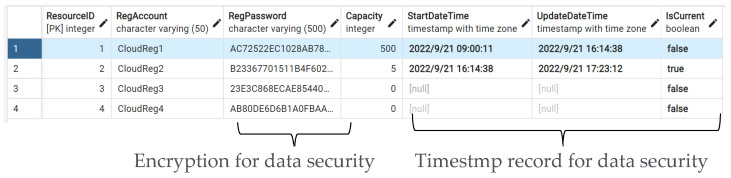
The results of the resource pool.

**Figure 12:**
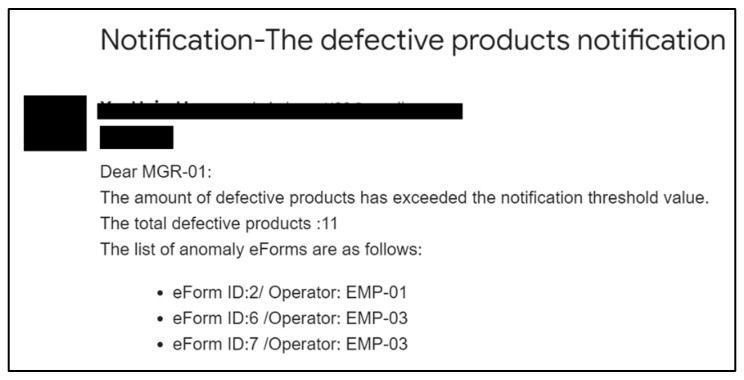
The result of automatically notification via the distributed emailing resource.

The automatic notification triggers when the real-time value exceeds the threshold value. When the data value achieves the threshold value or the predicted the potential defective event occurrs, then the data or the predicted event transmits the proposed resource-dispatching approach. Figure 10 shows the average system time with the 10-time repeated simulation; the mean of system time is 0.178 s, which means that the proposed method can quickly respond from the data to the user. Figure 11 shows that the registered third-party resources were stored in the resource pool for the proposed dispatching approach to be arranged in the data communication. The transmitted data obtained the arranged third-party emailing resource. This study organizes the transmitted data into notification content and the emailing of the notification using the arranged resource. Figure 12 shows that emailing can be correctly sent to the user using the arranged resource. One thousand simulation data points and seven emailing resources were applied in the system simulation and the data was arranged according to the corresponding emailing resource using the proposed resource dispatching approach.

## 6. Conclusions

This research collected e-Form data from a computerized sewing machine when initiating the manufacturing process, and the data had limited potential for further use in other applications. The primary issue that this study aimed to address was the use of current e-Form data from the computerized sewing machine to enhance data utilization for improving the maintenance efficiency of textile product manufacturing through intelligentization and automation. To intelligentize production, current data can be applied to defective product prediction using a machine learning approach. This study demonstrated that the ELAs used for overall performance evaluation had average accuracy. The results revealed that LightGBM had superior classification performance and computation time. Previous research has indicated that LightGBM has better accuracy than other gradient-boosting decision trees [33,36], and it is extensively applied for predicting industrial tools’ RUL [36]. The boosted ensemble method demonstrated high accuracy for the predictive problem [20]. These aforementioned studies support the results of this study. The bagging method attempts to reduce the error level due to the base classifier’s variance by voting on each model’s optimal performance [25,30]. The generalization ability of a training model refers to its ability to adapt to new and previously unseen data. The decision jungle is based on the bagging ensemble method; thus, it has the advantages of memory efficiency [32], good accuracy [37], and generalization for several tasks. This study uses the decision jungle based on the bagging approach to reinforce training the weak instance of the LightGBM model. The proposed approach outperforms the other ensemble approaches in terms of accuracy and computation time. To automatize production, transmission data streams between edge-side equipment and cloud-side communications are important for IoT advancement [38]. This study showed that the original data enabled analytics results to be correctly submitted to the cloud, third-party emailing resources to be arranged, and automatic notifications for PdM to be sent. The repeated system time simulation demonstrated that the proposed method could quickly respond to users with the correct data information. The case results show that the proposed approach can help with product quality maintenance and improve inspection efficiency in the production cycle by combining data analytics and cloud technologies. The main finding of this study was the promising advantages of using ELAs to predict defective textile products, enabling users to receive automatic notifications from the original data and analytics results by developing the proposed resource dispatching approach. Finally, PdM’s efficiency can be enhanced by integrating data analytics and systems in the textile product manufacturing process. This study was constrained by the sample size and the limited data attributes. In terms of sample size limitation, the proportion of data with the defective label is small in the original dataset used in this study. In Industry 4.0, the manufacturer replaces human operations with digital manufacturing technologies to avoid potential defective events. Therefore, a defective event is unlikely to occur in the mature development of the advanced manufacturing process. Collecting data with defective labels is difficult in the practical manufacturing site. This study used the data balance method to oversample the original dataset. The generated data with the defective label are similar, which reduces the difficulty of classification. In terms of limited data attributes, the dataset used in this study has only a few attributes from the specified textile production. The manufacturing parameters are fixed and simple, and the production feature is evident in certain conventional manufacturing processes. The solution space of the original dataset is simple, and it easily achieves excellent classification performance. Despite the limitations of the original dataset, all ELAs achieved outperformance in predicting the defective class of product in this study. Accordingly, the proposed method presented no significant outstanding performance. Future studies will continue collecting more real data with defective labels to address the similarity of oversampled data. In future studies, the authors will expand on these findings by analyzing different data sources from the other e-Forms, computerized machines, or manufacturing execution systems. This would help determine the proposed method’s generalization ability for complex data attributes.

## Figures and Tables

**Table 1:** The description of experiment environment.

Main Part	Specification
Central Processing Unit	Intel(R) Core(TM) i7-7660U CPU 2.50 GHz
Random Access Memory	8.00 GB
Hard disk	237 G
Power supply unit	127-watt power supply

**Table 2:** List of datasets.

The Attribute	Description	Type	Total Instances
FormId	The id of the work order form	String	500 non-defective: 96% defective: 4%
P_Width	The width of the finished product	Numeric	
P_Length	The length of the finished product	Numeric	
P_Height	The height of the finished product	Numeric	
P_EmpNo	employee number of operators	String	
P_Quality	The quality of the finished product	Binary (1 = non-defective, 0 = defective)	

**Table 3:** Results of mean and variance over repeated runs from 10-fold validation.

Evaluation Criteria (Variance/Standard Deviation)	The Proposed Approach	Random Forest	Decision Jungle	Light GBM	XGBoost
Accuracy	0.000/0.013	0.001/0.036	0.001/0.033	0.000/0.017	0.001/0.037
Precision	0.001/0.026	0.001/0.038	0.000/0.011	0.001/0.031	0.002/0.050
Recall rate	0.000/0.014	0.005/0.070	0.000/0.017	0.000/0.016	0.004/0.062
F1-Score	0.000/0.012	0.001/0.037	0.000/0.017	0.000/0.017	0.001/0.037

**Table 4:** The overall results of the ensemble learning based models.

Evaluation Criteria (Means of 10-Folds)	The Proposed Approach	Random Forest	Decision Jungle	Light GBM	XGBoost
Accuracy	0.983	0.966	0.980	0.981	0.965
Precision	0.979	0.977	0.980	0.976	0.970
Recall rate	0.987	0.960	0.973	0.986	0.965
F1-Score	0.983	0.966	0.976	0.981	0.965
MCC	0.966	0.940	0.953	0.960	0.936
Computing time	6.2 s	6.9 s	5.9 s	4.2 s	6.67 s

## Data Availability

Not applicable.
